# Supplementary material for: Resolving the full spectrum of human genome variation using Linked-Reads
Source: Genome Res. 2019 Apr;29(4):635–45. doi: 10.1101/gr.234443.118 (PMC6442396; doi:10.1101/gr.234443.118)
Supplement: Supplemental Material [file supp_gr.234443.118_Supplemental_longranger-2.2.zip › longranger-2.2.tar.bz2!/lr2.2-public/lib/stan/cmdstan/stan/doc/api/html/internal__math_8hpp.html]

Stan: src/stan/prob/internal\_math.hpp File Reference


|  |  |
| --- | --- |
|  | Stan  2.5.0  probability, sampling & optimization |


- Main Page
- Related Pages
- Namespaces
- Classes
- Files

- File List
- File Members

All Classes Namespaces Files Functions Variables Typedefs Enumerations Enumerator Friends Macros Pages

- src
- stan
- prob

Namespaces |
Functions

internal\_math.hpp File Reference

`#include <math.h>`  
`#include <boost/math/special_functions/gamma.hpp>`  
`#include <boost/math/special_functions/beta.hpp>`  
`#include <boost/math/special_functions/fpclassify.hpp>`

Go to the source code of this file.

|  |  |
| --- | --- |
| Namespaces | |
|  | stan |
|  | Probability, optimization and sampling library. |
|  | |
|  | stan::math |
|  | Matrices and templated mathematical functions. |
|  | |

|  |  |
| --- | --- |
| Functions | |
| double | stan::math::F32 (double a, double b, double c, double d, double e, double z, double precision=1e-6) |
|  | |
| void | stan::math::gradF32 (double \*g, double a, double b, double c, double d, double e, double z, double precision=1e-6) |
|  | |
| void | stan::math::grad2F1 (double &gradA, double &gradC, double a, double b, double c, double z, double precision=1e-6) |
|  | |
| void | stan::math::gradIncBeta (double &g1, double &g2, double a, double b, double z) |
|  | |
| void | stan::math::gradRegIncBeta (double &g1, double &g2, double a, double b, double z, double digammaA, double digammaB, double digammaSum, double betaAB) |
|  | |
| double | stan::math::gradRegIncGamma (double a, double z, double g, double dig, double precision=1e-6) |
|  | |


---

[ Stan Home Page ]

*© 2011–2014,
Stan Development Team.*
